# Bacterial and fungal coinfections in COVID-19 patients hospitalized during the New York City pandemic surge

**DOI:** 10.1017/ice.2020.368

**Published:** 2020-07-24

**Authors:** Priya Nori, Kelsie Cowman, Victor Chen, Rachel Bartash, Wendy Szymczak, Theresa Madaline, Chitra Punjabi Katiyar, Ruchika Jain, Margaret Aldrich, Gregory Weston, Philip Gialanella, Marilou Corpuz, Inessa Gendlina, Yi Guo

**Affiliations:** 1 Division of Infectious Diseases, Department of Medicine, Montefiore Medical Center, Albert Einstein College of Medicine, Bronx, New York; 2 Department of Pharmacy, Montefiore Medical Center, Albert Einstein College of Medicine, Bronx, New York; 3 Department of Pathology, Montefiore Medical Center, Albert Einstein College of Medicine, Bronx, New York; 4 Department of Pediatrics, Division of Infectious Diseases, Montefiore Medical Center, Albert Einstein College of Medicine, Bronx, New York

## Abstract

We observed bacterial or fungal coinfections in COVID-19 patients admitted between March 1 and April 18, 2020 (152 of 4,267, 3.6%). Among these patients, mortality was 57%; 74% were intubated; 51% with bacteremia had central venous catheters. Time to culture positivity was 6–7 days, and 79% had received prior antibiotics. Metallo-β-lactamase–producing *E. cloacae* coinfections occurred in 5 patients.

Few studies have addressed bacterial or fungal coinfections or the emergence of antimicrobial resistance in coronavirus disease 2019 (COVID-19) patients. More than 70% may receive antibiotics, but <10% experience coinfections.^1,2^ These patients have multiple risk factors for poor outcomes associated with nosocomial infections, such as critical illness, prolonged hospitalization, mechanical ventilation, and immune dysregulation.^1^ Given the mismatch between empiric prescribing and coinfection rates, recent World Health Organization guidelines recommend empiric antibiotics only for patients with severe COVID-19, using host factors and local epidemiology to drive antibiotic selection.^3^ We sought to characterize the microbiology of bacterial and fungal coinfections during the pandemic surge at our medical center with a focus on clinical outcomes, antimicrobial use, and antimicrobial resistance (AMR).

## Methods

We conducted a retrospective observational study of COVID-19 patients admitted between March 1, 2020, and April 18, 2020. Microbiology data were obtained from the laboratory information system (LIS). Patient demographics, central venous catheter status, ICU status, mechanical ventilation status, imaging, laboratory results, administered antibiotics per days of therapy (DOT), and disposition (admitted, discharged, deceased) were obtained from the electronic medical record. All cases were reviewed by an infectious diseases (ID) specialist to determine (1) the presence of true clinical coinfection and (2) the source. National Healthcare Safety Network (NHSN) criteria were used for central-line–associated bloodstream infections (CLABSI). Antibiogram data from March 1 to April 23, 2019 versus 2020 (institution-wide) and 2018–2019 (ICU-specific) were compared. Institutional review board approval was obtained (IRB no. 2020-11285).

Descriptive statistics were summarized using frequencies and percentages, or medians and interquartile ranges (IQRs). Bivariate analyses were conducted (χ^2^ or Fisher exact test). Analyses were conducted using SAS version 9.4 software (SAS Institute, Cary, NC). All statistical tests were 2-tailed and *P* values < .05 were considered significant.

### Inclusion criteria

All adult and pediatric patients with a positive SARS-CoV-2 PCR result and positive blood or respiratory culture (by matrix-assisted laser desorption/ionization) were analyzed. Cases were included if the positive PCR result and microbiology result occurred in the same or preceding admission (within 30 days).

### Exclusion criteria

Blood cultures positive for skin flora that did not grow in multiple cultures or on separate dates were excluded (ie, gram-positive bacilli, coagulase-negative staphylococci [CONS], micrococci, *Kocuria* spp). Respiratory cultures positive for yeast, normal oral or respiratory flora, mixed bacterial species, and skin flora were excluded. Patients with positive urine cultures alone without concurrent bacteremia were excluded.

## Results

### Patient demographics

In total, 152 distinct patients were analyzed among 4,267 COVID-19 patients admitted between March 1, 2020 and April 18, 2020 (3.6%). Of these, 32% of patients were Hispanic, 39% were non-Hispanic black, and 7% were white. Also, 89 patients (59%) were men, and the median age was 62 years (IQR, 52.5–72). Moreover, 33% had had preceding healthcare exposure defined as recent hospitalization, residence in a skilled nursing facility, or chronic hemodialysis.

In total, 99 patients (65%) were admitted to intensive care units (ICUs) and 112 patients (74%) received mechanical ventilation (in the ICU or ward). Overall, 86 patients (57%) died, 24 patients (16%) were discharged, and 42 patients (28%) were still admitted at the time of the analysis. Median length of hospitalization was 13 days (IQR, 6–21). In addition, 26 patients (17%) received biologics (eg, anakinra, tocilizumab, sarilumab, or leronlimab) or placebo and 44 patients (29%) received corticosteroids (Table 1).

In total, 91 patients (60%) had positive respiratory cultures, 82 patients (54%) had positive blood cultures, and 21 patients (14%) had both positive blood and respiratory cultures with the same or different organisms. In addition, 13 patients (9%) had polymicrobial cultures (Table 2).

**Table 1.** Demographics, Comorbidities, and Clinical Characteristics

	**N=152** **(distinct patients)**	
	n or median	% or IQR
**Demographics**		
**Age, years, median (IQR)**	62	52.5–72
**Sex**		
Female	63	41%
Male	89	59%
**Race**		
Hispanic	48	32%
Non-Hispanic black	60	39%
Non-Hispanic white	11	7%
Asian	9	6%
Other	12	8%
Unknown	12	8%
**Co-infection**		
Blood only	61	40%
Respiratory only	70	46%
Both blood and respiratory	21	14%
**Comorbidities**		
Charlson Comorbidity Score	2	1–4
Immunocompromised*	84	55%
**Medications Received for COVID-19**		
Biologics**	26	17%
Acute steroid use	44	29%
**Outcomes**		
Length of stay, days	13	6–21
Still admitted at time of analysis	42	28%
Discharged alive	24	16%
Deceased	86	57%

*Immunocompromised includes chronic diabetes, HIV, hepatitis C, active malignancy, organ transplant, rheumatologic disease, or chronic receipt of immunosuppressive medications. **Patients received anakinra, tocilizumab, sarilumab, or leronlimab either through randomized clinical trial or compassionate use; unknown if trial patients received placebo or study medication.

**Table 2.** COVID-19 Patients with Positive Respiratory and/or Blood Cultures

	**Respiratory** **N=91**		**Blood** **N= 82**	
	n or median	% or IQR	n or median	% or IQR
**Microbiology**				
**Timing of Culture Results**				
Time between positive bacterial culture and SARS-CoV-2 PCR results, days	6	2–8	7	3–14
Patients with positive bacterial culture result prior to positive SARS-CoV-2 PCR result	4	4%	17	22%
Patients with positive bacterial culture and SARS-CoV-2 PCR results on same day	2	2%	22	26%
**Multidrug-resistant Organism**	17	19%	7	9%
**Documented Catheter of Any Type on Earliest Date of Bacteremia (Excluding Foley)**				
**Total Number of Catheter**	–	–	44	54%
Hemodialysis catheter	–	–	13	16%
Central venous catheter	–	–	37	45%
Peritoneal dialysis catheter	–	–	4	5%
**Source of Bacteremia**	–	–		
Gastrointestinal	–	–	6	7%
Genitourinary	–	–	7	9%
Catheter	–	–	19	23%
Respiratory	–	–	11	13%
Oral pharyngeal	–	–	2	2%
Skin	–	–	5	6%
Multiple sources	–	–	25	30%
Other	–	–	2	2%
Unknown	–	–	5	6%
**Clinical Characteristics**				
**Initial Chest X-ray**				
Bilateral opacities	76	84%	–	–
Unilateral opacities	6	7%	–	–
Interstitial	4	5%	–	–
**Pneumonia on Initial Chest X-ray**	–	–	72	88%
**Critical Care Admission**	85	93%	33	40%
**Ward Admission Only**	6	7%	39	48%
**Emergency Department Only**	0	0%	10	12%
**Intubated**	86	95%	46	56%
**Maximum Lab Values, median**				
WBC, k/uL	20.6	15.9–29.7	15.7	10.9–24.7
CRP, mg/dL	31.2	20.9–41.8	19.3	0–37.3
PCT, ng/mL	1.9	0.4–10.9	0.8	0–9.9
**Outcomes**				
Length of stay, days	15	9–21	12	3–24
Still admitted at time of analysis	30	33%	23	28%
Discharged alive	8	9%	17	21%
Deceased	53	58%	42	51%

### Respiratory coinfections

**Fig. 1.**
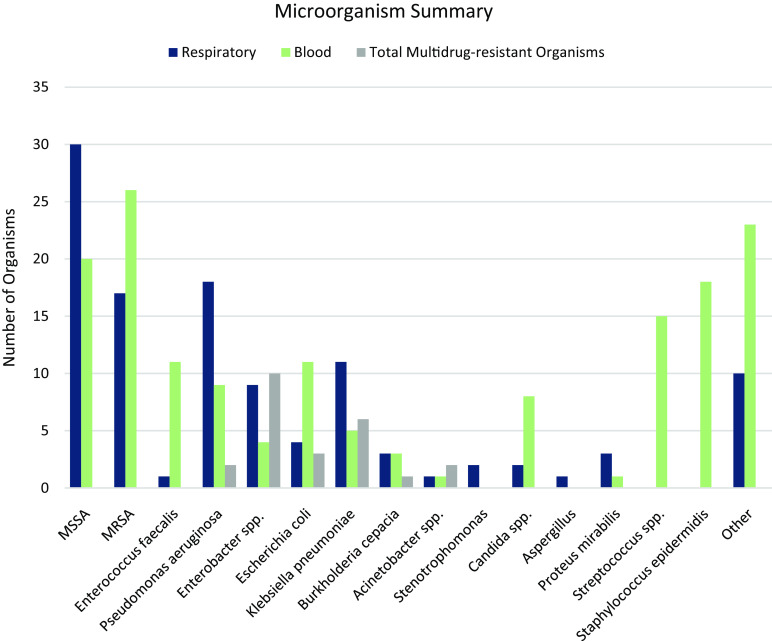
Microorganism summary. Note. *Abbreviations: MSSA, Methicillin-sensitive Staphylococcus aureus; MRSA, Methicillin-resistant Staphylococcus aureus; spp., species Other microorganisms include: *Stenotrophomonas maltophilia, Serratia marcescens, Actinomyces neuii, Corynebacterium afermentans, Corynebacterium matruchotii, Rothia mucilaginosa, Elizabethkingia meningoseptica, Blautia coccoides, Escheria vulneris, Prevotella disiens, Fusobacterium necrophorum, Bacteroides ovatus group, Bacteroides thetaiotaomicron, Fusobacterium nucleatum, Achromobacter xylosoxidans, Chryseobacterium gleum, Citrobacter koseri, Haemophilus parainfluenzae, Serratia marcescens*

Among the 91 patients with positive respiratory cultures, 112 isolates were identified (2). The 5 most commonly identified organisms were *S. aureus* (44%), *P. aeruginosa* (16%), *Klebsiella* spp (10%), *Enterobacter* spp (8%), and *E. coli* (4%) (Fig. 1). Moreover, 17 gram-negative isolates (15%) were multidrug resistant, defined as resistance to at least 1 agent in at least 3 different antibiotic classes. Among them, 6 (5%) were carbapenem-resistant *Enterobacteriaceae* (CRE). The median time between SARS-CoV-2 PCR result and positive respiratory culture was 6 days (IQR, 2–8 days). Most patients were admitted to ICUs (93%) and were intubated (95%). In addition, 4 patients (4%) had positive respiratory cultures ≥1 day prior to the SARS-CoV-2 result, all of whom were admitted from long-term care facilities.

### Bloodstream coinfections

Among the 82 patients with positive blood cultures, 155 isolates were identified (2). The median time to bacteremia was 7 days (IQR, 3–14 days). Also, 44 patients (54%) had a documented central venous catheter at the time of bacteremia. The following sources of infection were determined by an ID specialist: catheter (23%), respiratory (13%), genitourinary (9%), gastrointestinal (6%), or multiple (30%) (2). The NHSN CLABSI criteria were met in 13 of 19 cases (68%), and the remainder were considered clinical CLABSIs or secondary bloodstream infections.

The most frequently isolated organisms were *Staphylococcus aureus* (30%), *S. epidermidis* (12%), *Streptococcus* spp (10%), *Enterococcus* spp (7%), *Escherichia coli* (7%), *Pseudomonas aeruginosa* (6%), *Candida* spp (5%), *Klebsiella* spp (3%), and *Enterobacter* spp (3%) (Fig. 1). The study cohort included 8 candidemia patients; 7 patients had central venous catheters. Also, 7 gram-negative bloodstream isolates (8.5%) were multidrug resistant (MDR), of which 4 (5%) were CRE. Candidemia was observed in 8 COVID-19 patients and in 31 patients overall in this study period (vs 25 in 2019).

Of 82 patients, 17 (21%) were initially bacteremic then had a subsequent positive SARS-CoV-2 PCR result. Bacteremic episodes occurred during the COVID-19 admission (n = 12, 70%) or a prior admission (n = 5, 30%). In addition, 22 patients (27%) had a concurrent positive SARS-CoV-2 result and bacteremia with a variety of gram-positive and gram-negative bacteria (eg, MSSA, MRSA, *P. aeruginosa*, *E. coli*, *Streptococcus* spp, etc).

### Antibiotic use

Of 5,853 COVID-19 patients admitted between March 1 and May 31, 2020, 4,130 patients (71%) received at least 1 antibiotic dose of the following agents: doxycycline, azithromycin, levofloxacin, ciprofloxacin, ceftriaxone, cefepime, intravenous vancomycin, and piperacillin/tazobactam. Also, 120 patients in this study (79%) had antibiotic exposure in the 30 days preceding positive microbiology. All 21 patients (100%) with MDR infections had received prior antibiotics compared to 99 patients (65%) without MDR infections (*P* = .01).

Overall, 149 patients in the study (98%) received antibiotics at some point during their COVID-19 hospitalization. The median antibiotic days of therapy (DOT) was 8.5 days (IQR, 5–14); 12 days (IQR, 7–20) in patients with multidrug resistance, and 8 days (IQR, 4–14) in patients without (*P* = .21). In addition, 107 patients (70%) received >3 antibiotic classes (β-lactams, glycopeptides, macrolides, or tetracyclines).

### Local epidemiology and AMR

Institution-wide antibiogram data were compiled for March 1–April 23, 2019, versus 2020 for *P. aeruginosa*, *E. coli*, *K. pneumoniae*, and *S. aureus* (≥30 clinical isolates). *Klebsiella pneumoniae* susceptibility to cephalosporins, ciprofloxacin, and meropenem decreased by >10% between 2019 and 2020 (*P* < .05). In total, 12 Enterobacteriaceae isolates were resistant to carbapenems in 2019 (vs 17 in 2020). Also, 5 patients admitted between March 28 and April 22, 2020 developed infection with New Delhi metallo-β-lactamase (NDM)–producing *E. cloacae* isolated in respiratory cultures alone (n = 2) or blood and respiratory cultures (n = 3). All were admitted from the community, without international healthcare exposure, and 4 of 5 succumbed to septic shock.

The March 1–April 23, 2020, antibiogram comparison to the institutional ICU antibiogram for 2018–2019 revealed a >10% decline in the following susceptibilities: (1) *K. pneumoniae* versus aztreonam, cefepime, ceftriaxone, ciprofloxacin, gentamicin, meropenem, piperacillin/tazobactam, and tobramycin; (2) *E. cloacae* vs. aztreonam, ceftriaxone, meropenem, piperacillin/tazobactam; and (3) *P. aeruginosa* versus amikacin. Increases in susceptibility >10% were observed for *P. aeruginosa* versus meropenem and *E. cloacae* versus gentamicin. There were 279 ICU-specific *S. aureus* isolates in 2018–2019 (60% MSSA) versus 151 institution-wide from March 1 through April 23, 2020 (65% MSSA). The median length of stay was 15 days for patients with MDR infection versus 13 days for patients without MDR infection (*P* = .09). Moreover, 15 patients with MDR infection (71%) had died at the time of analysis versus 70 patients without MDR infection (54%; *P* = .12).

## Discussion

We observed widespread empiric antibiotic use throughout the pandemic and clinically relevant bacterial and fungal coinfections in patients with advanced COVID-19 and multiple risk factors for nosocomial infection (mechanical ventilation, central venous catheters, treatment with corticosteroids or biologics, and prolonged hospitalization). Although comparative NYC rates of pandemic antibiotic use and nosocomial infections were not available, the Bronx had the highest rates of COVID-19 hospitalizations and deaths.^4,5^ Therefore, these observations are expected to a certain extent.^2^ Blacks and Hispanics comprised 71% of our study population, but we were unable to determine the impact of race on mortality due to coinfection.

Due to strain experienced by the health systems at surge capacity, attention was likely diverted away from monitoring for excess antimicrobial use and nosocomial infections.^2^ We are particularly concerned about the number of candidemias that met NHSN CLABSI criteria. The potential impact on healthcare-associated infection rates is a significant concern for hospitals.^6^ Coinfections reported during past coronavirus pandemics were also healthcare associated.^2^

Blood cultures positive for skin flora were excluded from analysis, but the number of coagulase-negative staphylococci bacteremia cases significantly increased from 110 to 269 over the same period in 2019 versus 2020, suggesting a higher rate of blood culture contamination. Although this finding reflects an absolute increase in number of specimens sent, formal observations of blood-culturing technique, and catheter insertion and maintenance procedures are needed to evaluate fidelity to prepandemic infection prevention bundles.

The clinical presentation of severe COVID-19 may be indistinguishable from bacterial or fungal sepsis, which is likely driving excess antimicrobial use.^1,7^ Like earlier studies, we observed a significant mismatch of antibiotic use (71%) versus coinfections (3.6%).^2,6^ Moreover, 79% of coinfected patients received antibiotics in the 30 days preceding positive cultures and 98% received them during the index COVID-19 hospitalization. In the latter group, empiric or targeted antibiotics were administered for a median of 8.5 days, and 70% of patients received >3 antibiotic classes. Therefore, antimicrobial stewardship programs have a major contributory role in the pandemic response with rational empiric antibiotic guidelines.^1,2,8^ We suggest use of “real-time” institutional antibiograms to guide protocol development.

To our knowledge, this is the first description of the microbiology and clinical outcomes of bacterial and fungal coinfections during the NYC COVID-19 pandemic surge. Clinical coinfections were confirmed by an ID specialist and contaminants were excluded. Goyal et al^9^ reported a higher rate of bacteremic patients at a neighboring NYC institution (19 of 338, 5.6%) but did not report specific microbiology or contamination rate. Antibiogram data comparing 2018–2019 and 2020 revealed a significant decline in *Enterobacteriaceae* susceptibilities to multiple antibiotics, potentially due to selective antibiotic pressure. Although most infections occurred after initial COVID-19 diagnosis (70%), 16% of patients had a concurrent positive SARS-CoV-2 PCR and microbial culture with a variety of bacteria. Furthermore, in 2018–2019, there were 279 ICU *S. aureus* clinical isolates versus 151 during the <8 week study period, suggesting a proportionally higher number of *S. aureus* infections during the pandemic. Further study is warranted to determine increased susceptibility to *S. aureus* and other pathogens similar to that observed during past influenza A pandemics.^10^

Overall, 70 patients (46%) received either corticosteroids or biologics; however, our study was not designed to detect differences in infection rates or types of pathogens among patients who did or did not receive immunosuppressive medications.

This study has several limitations. This is a single-center observational report of only 152 patients with no comparison to matched controls without secondary infection, which is needed to truly assess differences in AMR and clinical outcomes. AMR due to the pandemic may be exacerbated in cities with pre-existing high prevalence; therefore, our results may not be generalizable to other regions.^1^ Urine culture results were not reviewed unless patients had concurrent bacteremia. At the onset, respiratory cultures were obtained on a limited basis due to potential for aerosolization; therefore, the true number of concurrent bacterial pneumonias remains unknown. The study was not designed to determine the cause of secondary infection among the numerous possibilities (eg, disruption of host immunity, hospital acquisition, immunosuppressive medications, provider practice changes, etc). Regardless, we suggest reinforcement of infection prevention and stewardship best practices.

In conclusion, our study confirms widespread antibiotic use in most hospitalized COVID-19 patients at our medical center. Bacterial and fungal coinfections occurred in <5% but are of significant concern due to their occurrence in the most vulnerable patients. In addition, we observed worsening Enterobacteriaceae susceptibility profiles emerging during the brief study period compared to antibiogram data from 2018 to 2019. The pandemic has highlighted the need for close collaboration between stewardship and infection prevention programs to monitor for nosocomial infections, excess antibiotic use, and multidrug resistance.
